# The Flavor Characteristics and Metabolites of Three Commercial Dried Jujube Cultivars

**DOI:** 10.3390/foods13081193

**Published:** 2024-04-14

**Authors:** Yuyao Jia, Chao Wang, Ying Zhang, Wenkai Deng, Yicai Ma, Juanfang Ma, Gang Han

**Affiliations:** College of Forestry, Northwest A&F University, Xianyang 712100, China; jyy18009072697@163.com (Y.J.); wangchao_916@163.com (C.W.); tclwysl@126.com (Y.Z.); dengy96@163.com (W.D.); mayicai1002@163.com (Y.M.); m_juanfang@163.com (J.M.)

**Keywords:** jujube, flavor, nutrition, metabolomic

## Abstract

To understand the flavor and metabolite differences between the three commercial dried jujube cultivars *Huizao* (HZ), *Hamazao 1* (HMZ), and *Qiyuexian* (QYX), their soluble sugars, organic acids, volatiles, and metabolites were systematically investigated. The results show that sucrose and malic acid were the main soluble sugar and organic acids contained in these dried jujubes, respectively. Sucrose (573.89 mg/g DW) had the highest presence in HZ, and the total sugar content (898.33 mg/g DW) was the highest in QYX. Both of these had a low total acid content, resulting in relatively high sugar–acid ratios (105.49 and 127.86, respectively) compared to that of HMZ (51.50). Additionally, 66 volatile components were detected in the 3 jujubes. These mainly included acids, aldehydes, esters, and ketones (90.5–96.49%). Among them, (E)-2-nonenal, (E)-2-decenal, heptanal, decanal, nonanal, and octanal were identified as the key aromatic substances of the dried jujubes, and their contents were the highest in HMZ. Moreover, 454 metabolites were identified, including alkaloids, amino acids, flavonoids, lipids, nucleotides, and terpenoids. The highest contents of flavonoids (5.6%) and lipids (24.9%) were detected in HMZ, the highest contents of nucleotides (10.2%) and alkaloids (27%) were found in QYX, and the contents of saccharides (5.7%) and amino acids (23.6%) were high in HZ. Overall, HZ, HMZ, and QYX significantly differ in their flavor and nutrition. HZ tastes better, HMZ is more fragrant, and QYX and HMZ possess higher nutritional values.

## 1. Introduction

Jujube (*Ziziphus jujuba* Mill.), a light- and heat-loving, drought-tolerant tree species with a cultivation and utilization history spanning over 7000 years, is native to China and widely cultivated in multiple regions, such as Europe, southern and eastern Asia, and Australia [1,2]. Jujube is an important economic fruit tree in the Rhamnaceae family [3,4]. Additionally, China possesses the largest jujube planting area and jujube fruit output in the world. The main production areas of jujube are divided into the traditional middle and lower reaches of the Yellow River, where fresh jujube is produced, and the Northwest Desert arid system area, where dry jujube is produced [5]. Xinjiang has low rainfall, which can prevent the cracking of jujubes during ripening and reduce the impact of rain on the quality of jujube fruits. It also has sufficient sunshine, a wide temperature difference between day and night, and extremely low rainfall during the jujube fruit maturation period, which helps metabolites to accumulate in jujubes, improving their fruit quality and creating ideal conditions for the production of dried jujube fruit. Due to these superior natural conditions, Xinjiang has become the largest and most important jujube production area, where many inland fine jujube cultivars have been introduced and are being cultivated. In recent years, over 460,000 hectares of jujube have been planted in Xinjiang, which has become the largest dried-jujube-fruit-producing area in China (accounting for 60%) [5,6,7].

*Huizao* (HZ), *Hamazao 1* (HMZ), and *Qiyuexian* (QYX), originating from Xinzheng in Henan Province, Heyang in Shaanxi Province, and Yongji in Shanxi Province, respectively, are the three most significant jujube cultivars introduced to Xinjiang. With a soluble solid content of more than 28% and an edibility rate of more than 94%, their fresh fruits are juicy, sweet, and crisp, making them superior cultivars of jujube for fresh consumption [8,9,10]. However, they are primarily grown as dried-use jujube cultivars in Xinjiang. In detail, HZ was introduced in 1987 and has the largest cultivation area; QYX was introduced over the last ten years and has a specific cultivation scale; and HMZ is a highly promising cultivar that is currently being promoted in Xinjiang. However, the changes that have occurred in their traits and quality after their introduction have not been studied.

Compared to fresh jujube fruits, dried jujube fruits have a longer shelf life and are conducive to long-term preservation and utilization [11,12]. In addition, the taste and smell of dried jujube are improved by reducing the moisture content, increasing the sugar concentration, and producing uniquely scented compounds [13,14]. Dried jujube is rich in amino acids, polysaccharides, organic acids, phenols, vitamins, and mineral nutrients. It can be eaten directly and is commonly used in food processing, such as for jujube chips, jujube paste, jujube vinegar, and jujube wine [15,16,17]. Jujube is also used as a traditional Chinese medicine, with many pharmacological benefits, such as aiding sleep, the protection of the liver and nerves, and anti-cancer effects. In addition to this, jujubes also have antiseptic, anti-inflammatory, antioxidant, and intestinal protection effects [18,19,20]. These beneficial effects are closely related to the natural active ingredients they contain. People are often willing to pay for the health benefits of jujubes. In general, the development and utilization value of dried jujube in foods and medicines is closely related to its rich plant volatile components (VOCs) and metabolites.

The VOCs and metabolites found in jujube fruits have been reported by certain researchers in recent years. Zhu and Xiao [14] identified 37, 37, and 35 volatiles in the dried fruits of *Jinsixiaozao*, *Youzao* and *Yuzao*, respectively, and found that hexanal, (E)-2-octenal, β-damascenone, ethyl hexanoate, 3-mercaptohexyl acetate, and 2,5-dimethylpyrazine are the key odor-active compounds. A total of 33 VOCs were identified among 15 fresh jujube cultivars. Among them, hexanal, (E)-2-hexanal, nonanal, and n-decanoic acid were found to be the major volatile compounds of fresh jujube fruits [21]. The metabolites of *Jinsixiaozao* were investigated, which mainly included 66 lipids, 55 flavonoids, 42 terpenoids, 42 amino acids and their derivatives, and 38 alkaloids. Among these, most of the amino acids and triterpenes gradually accumulate during development, while the presence of flavonoids decreases gradually during development [22]. Flavonoids have also been proven to be important secondary metabolites that affect the color of jujube peels and jujube leaves [23,24]. Although many studies on the VOCs and metabolites of jujube fruits have been reported, systematic research is lacking on the sugar, acid, and flavor-related substances affecting the taste of dried jujube and the nutrients affecting their nutrition. The use of these data for the comprehensive utilization of different jujube varieties is especially under-reported.

In this work, three commercial jujube cultivars planted in Xinjiang (HZ, QYX, and HMZ) were used as the test materials. Their soluble sugars, organic acids, volatile components, and metabolites were isolated and identified using high-performance liquid chromatography (HPLC), headspace solid-phase microextraction–gas chromatography-mass spectrometry (HS-SPME-GC-MS/MS), and ultra-performance liquid chromatography tandem-mass spectrometry (UPLC-MS/MS), respectively. The key taste components, aroma-active compounds, differential metabolites, and potential differential metabolic pathways from the three jujube cultivars were systematically investigated to reveal the main variations in flavor and nutritional values among these varieties. Hence, the research data described here will contribute to the evaluation of the flavor and nutritional qualities of different jujube cultivars, thus providing a theoretical basis for the development of dried jujube products with desirable flavor and nutritional qualities.

## 2. Materials and Methods

### 2.1. Plant Materials and Chemicals

The dried jujube fruit materials of three cultivars, namely, HZ, QYX, and HMZ, were collected from the experimental jujube garden in Hotan, Xinjiang, China. In late October, the period when jujube fruit is fully ripe (the end of the ripening stage), we randomly selected 5 healthy, growing fruit trees (more than three years old) of each variety. With 40 jujubes collected per tree, we randomly selected 10 fruits from the upper layer of each plant in the eastern, western, southern, and northern directions, for a total of 200 jujubes of each variety, and collected them in a single day. We brought them back to the laboratory and left them to shade-dry under indoor conditions (temperature of 15~25 °C; relative humidity of 45~70%) until the moisture content was about 23%. Then, each variety of dried jujube fruits was divided into three parts (i.e., three biological repetitions), pitted, put into a freeze dryer (Scientz-100F, Ningbo, China) for vacuum freeze-drying, crushed (30 Hz, 1.5 min) into powder with a mixer mill (MM 400, Retsch, Haan, Germany), and then stored at −80 °C until further use.

Chemicals, such as sodium hydroxide (NaOH), potassium phosphate monobasic (KH_2_PO_4_), concentrated sulfuric acid (H_2_SO_4_), and anthrone, were purchased from Sinopharm Chemical Reagent Co. (Shanghai, China). Standard solutions, including fructose, glucose, sucrose, malic acid, citric acid, quinic acid, and succinic acid, were obtained from Yuanye Biotechnology, Ltd. (Shanghai, China). Acetic acid, acetonitrile, methanol, and ethanol (HPLC grade) were obtained from Merck (Darmstadt, Germany).

### 2.2. Determination of Sugars and Acids in Different Jujube Cultivars

The contents of total sugar (TSC) and total acid (TAC) were determined using anthrone colorimetry and acid–base titration, respectively. The sugar–acid ratio (SAR) was calculated using the equation of SAR = TSC/TAC.

The soluble sugars (sucrose, fructose, and glucose) and organic acids (malic acid, citric acid, succinic acid, and quinic acid) were determined using the HPLC-RI method and the HPLC-UV method, respectively [25]. Briefly, dried jujube powder (200 mg) was extracted using 7.5 mL of 80% ethanol in a 70 °C water bath (DK-926, Shanghai, China) for 30 min, and then centrifuged at 8000 r/min (5424, Eppendorf, Hamburg, Germany) for 10 min. The obtained solution was then diluted to 25 mL, dried at 55 °C, redistilled and dissolved by adding 10 mL water, and then filtered with a 0.45 μm filter head. The filtrate was saved for testing.

The soluble sugars of the filtrate were measured with a chromatographic column (4.6ID × 150 nm, COSMOSIL Packed Column 5NH2-MS) and RI Detector L-2490, using sucrose, fructose, and glucose as the standards. The instrument parameters were set as follows: we chose a column temperature of 35 °C and detection temperature of 35 °C. The mobile phase was acetonitrile/water (80:20), which was filtered through a 0.22 μm membrane, and then subjected to ultrasonic degassing for 30 min. The flow rate was 1 mL/min, with an injection volume of 10 μL.

The organic acids of the filtrate were measured with a chromatographic column (4.6 × 250 mm, EOOSIL HPLC COLUMN C18) and UV Detector L-2400, using malic acid, citric acid, succinic acid, and quinic acid as the standards. The parameters were as follows: we chose a column temperature of 30 °C, a detection wavelength of 210 nm, and a detection temperature of 30 °C. The mobile phase was a KH_2_PO_4_ solution (0.04 mol/L) with an adjusted pH = 2.4, which was filtered through a 0.22 μm filter membrane and then subjected to ultrasonic degassing for 30 min. The flow rate was 0.5 mL/min, with an injection volume of 10 μL.

#### 2.2.1. Sugar Standard Curve Preparation

We dissolved 100 mg of sucrose, fructose, and glucose into 10 mL of ultrapure water to form a mother solution. We diluted the mother solution with ultrapure water into a series of mixed solutions of 0.05, 0.1, 0.25, 0.5, and 1.0 mg/mL.

$$Content\;of\;each\;sugar=\frac{Concentration\;of\;each\;component\;(mg/mL)\times constant\;volume\;(mL)}{Sample\;quality\;(g)}$$

#### 2.2.2. Acid Standard Curve Preparation

Standard curve preparation: 100 mg of malic acid, succinic acid, citric acid, and quinic acid were dissolved in 10 mL of ultrapure water to form a mother solution, and the mother solution was diluted with ultrapure water to form a series of mixed solutions of 0.05, 0.1, 0.25, 0.5, and 1.0 mg/mL.

$$Content\;of\;each\;acid=\frac{Concentration\;of\;each\;component\;(mg/mL)\times constant\;volume\;(mL)}{Sample\;quality\;(g)}$$

### 2.3. Determination of VOCs in Different Jujube Cultivars

Jujube powder (1 g) was weighed, placed in a headspace vial, and incubated at 60 °C for 15 min. The VOCs were absorbed using headspace solid-phase microextraction (HS-SPME), and then analyzed with a gas chromatography tandem mass spectrometer (GC-MS/MS, Agilent 7890B-7000C, Agilent, Santa Clara, CA, USA) equipped with an HP-5MS capillary column (30 m × 250 μm × 0.25 μm). The volatile components of the three varieties of jujubes were tested for esters, acids, alcohols, aldehydes, ketones, and so on. The main parameter settings (sample amount, extraction time, and extraction temperature) and the qualitative and quantitative determination processes of the VOCs were in line with those of our previous study [25].

### 2.4. Relative Odor Activity Value (ROAV) Calculation of VOCs

The ROAVs were calculated according to ROAV_i_ = 100 (C_i_/C_max_) (T_max_/T_i_), where T_i_ and C_i_ are the thresholds and relative content of an arbitrary VOC, and T_max_ and C_max_ are the threshold and relative contents of the component with the greatest aroma contribution, respectively [26]. The threshold values were taken from the existing literature on water.

### 2.5. Widely Targeted Metabolomic Analysis of Different Jujube Cultivars

#### 2.5.1. Sample Preparation and Metabolite Extraction

Dried jujube powder (100 mg) was extracted overnight at 4 °C using 1.0 mL 70% aqueous methanol. Following centrifugation at 10,000× *g*/min for 10 min, the extracts were absorbed (CNWBOND Carbon-GCB SPE Cartridge, 250 mg, 3 mL; ANPEL, Shanghai, China) and filtrated (SCAA-104, 0.22 μm pore size; ANPEL, Shanghai, China). Then, we used ultra-performance liquid chromatography (UPLC, Shim-pack UFLC SHIMADZU CBM30A system, Kyoto, Japan) with a tandem mass spectrometer (MS/MS, Applied Biosystems 6500 Q TRAP, Kyoto, Japan) to analyze the extracts.

#### 2.5.2. HPLC Conditions and ESI-Q TRAP-MS/MS Analysis

The *HPLC* analytical conditions were in accordance with the study of Chen et al. [27]. LIT and triple quadrupole (QQQ) scans were acquired using a triple quadrupole–linear ion trap mass spectrometer (Q TRAP; API 6500 Q TRAP LC/MS/MS System) equipped with an ESI Turbo Ion-Spray interface, operating in a positive ion mode and controlled with Analyst 1.6.3 software (AB Sciex, Framingham, MA, USA). The ESI source operation parameters were as follows: the ion source was a turbo spray; the source temperature was 500 °C; the ion spray voltage (IS) was 5500 V; the ion source gas I (GSI), gas II (GSII), and curtain gas (CUR) were set to 55, 60, and 25.0 psi, respectively; and the collision gas (CAD) was high. Instrument tuning and mass calibration were performed with 10 and 100 μmol/L polypropylene glycol solutions in the QQQ and LIT modes, respectively. The QQQ scans were acquired with multiple-reaction monitoring (MRM) experiments, with the collision gas (nitrogen) set to 5 psi. The DP and CE were determined for individual MRM transitions with further DP and CE optimization. A specific set of MRM transitions was monitored for each period according to the metabolites eluted within this period [27].

#### 2.5.3. Qualitative and Quantitative Determination of Metabolites

The mass spectrum data were processed with Analyst 1.6.3 software based on the Metware database (MWDB) (Wuhan Metware Biotechnology Co., Ltd., Wuhan, China). To ensure the accuracy of the metabolite annotation, we first excluded the isotope signals and repetitive signals, such as K^+^, Na^+^, and NH_4_^+^ ions, during the analysis [27]. Metabolite quantification was accomplished using the MRM mode of the QQQ MS. The detailed operation process is described in previous studies [28,29].

### 2.6. Statistical Analysis

All the samples used in this experiment were independent of each other. Firstly, IBM SPSS Statistics (Version 26.0, Chicago, IL, USA) was used to perform a normality test and homogeneity of variance test on the sample data, with *p* > 0.05 (normality test) and *p* > 0.05 (homogeneity of variance test) indicating that the samples conformed to the normal distribution and that the variance homogeneity test was passed. Then, we performed one-way analysis of variance (ANOVA) and Student’s *t*-test on all the data using IBM SPSS Statistics (Version 26.0, Chicago, IL, USA). Orthogonal partial least-squares discriminant analysis (OPLS-DA) and permutation tests (200 replicates) were performed using SIMCA-P 14.1 software (Version 14.1, Umetrics AB, Umea, Sweden). The differential metabolites were selected based on their variable importance in the projection (VIP) values > 1 and Log2FC > 1 (upregulated) or <−1 (downregulated), and then matched in the KEGG database and Metabolites Biological Role 2.0 to perform pathway enrichment analysis. Finally, Origin 2021 (Origin Lab Inc., Northampton, MA, USA) and TBtools (Version 1.09, Guangzhou, China) were used to render graphics.

## 3. Results and Discussion

### 3.1. Main Soluble Sugars and Organic Acids of Different Jujube Cultivars

**Figure 1:**
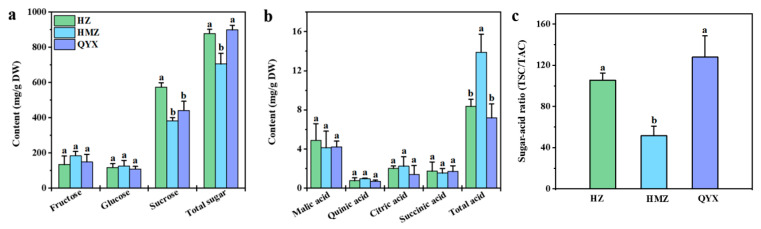
(**a**) Soluble sugars, (**b**) organic acids, and (**c**) sugar–acid ratio (TSC/TAC) in different dried jujube cultivars. Different lowercase letters indicate significant statistical differences (one-way ANOVA, *p* < 0.05) between different dried jujube cultivars.

Soluble sugars and organic acids are the main components that affect the traditional taste of fruits. Among them, soluble sugar is a key factor in determining the fruits’ sweetness [30]. Information on the soluble sugars of different dried jujube cultivars is shown in Figure 1a. The main sugars produced by dried jujube are fructose (155.88 mg/g DW), glucose (117.24 mg/g DW), and sucrose (466.07 mg/g DW), which account for 77.79–98.22% of all sugars. Sucrose, fructose, and glucose were also reported to be the main sugars of Shandong jujubes [31]. A previous study showed that sucrose was the most abundant soluble sugar in *Junzao* [32]. In this work, the sucrose content (573.89 mg/g DW) in HZ was the highest. The TSC of the three varieties showed the following trend: QYX (898.33 mg/g DW) > HZ (878.49 mg/g DW) > HMZ (705.71 mg/g DW).

An important indicator of fruit flavor quality, the sour taste of fruits comes from their organic acids [33]. As shown in Figure 1b, malic acid (4.41 mg/g DW) was found to have highest level of organic acid in the dried jujube fruit. Previous research has revealed that malic acid is also abundant in *Muzao* and *Junzao* [34,35]. Moreover, we found that the TAC was significantly different among the three jujube cultivars, with HMZ (13.90 mg/g DW) > HZ (8.36 mg/g DW) > QYX (7.18 mg/g DW).

Therefore, the high TSC and low TAC in HZ and QYX resulted in a higher sugar–acid ratio in HZ (105.49) and QYX (127.86) than in HMZ (51.50) (Figure 1c), which may explain why HZ and QYX taste better than HMZ.

### 3.2. VOC Profiles of Different Jujube Cultivars

**Figure 2:**
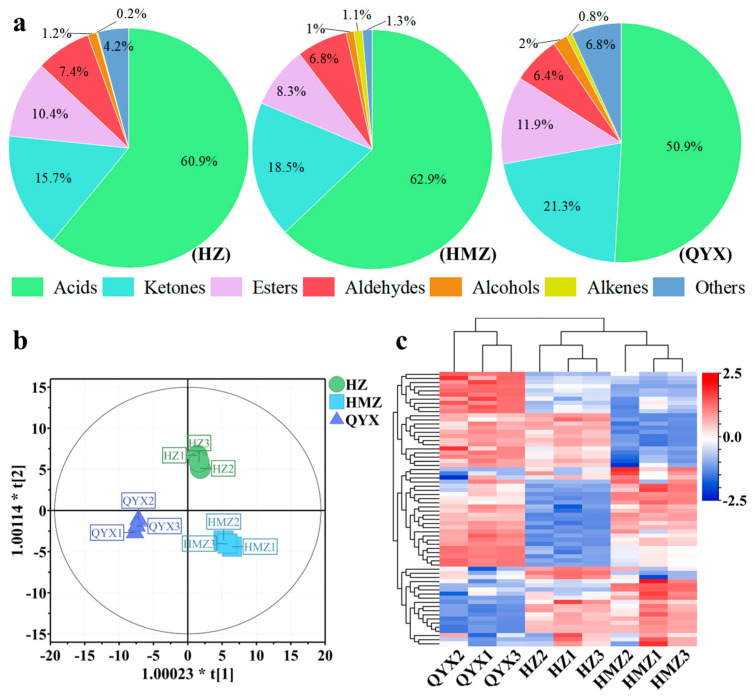
(**a**) Pie chart showing proportions of 7 VOC categories. (**b**) OPLS-DA score plot. (**c**) HCA of VOCs of different dried jujube cultivars.

Aromas are composed of a large number of volatile components. They produce short-term and integrated physiological sensations in people, mainly by stimulating the human olfactory and taste organs, thus affecting the human evaluation of the flavor of fruits [36]. Relevant studies have found that the volatile components of jujube mainly include esters, acids, alcohols, aldehydes, and ketones [21]. In this work, 66 volatile compounds were identified and classified into 7 categories, including 14 acids, 6 alcohols, 19 aldehydes, 2 alkenes, 7 esters, 10 ketones, and 8 other VOCs. Among them, the acids, aldehydes, esters, and ketones accounted for 90.5–96.49% of the total (Figure 2a). Acids, aldehydes, and esters were also identified as the dominant volatile categories of *Junzao*, *Hamidazao* and *Dongzao* [37,38]. It was revealed that more volatile components were produced in dried *Huizao* (64) compared to fresh versions (36), resulting in a richer flavor in dried jujubes than fresh jujubes [37].

In this work, HZ accumulated more aldehydes (7.42%), HMZ had the highest content of acids (62.88%) and alkenes (1.1%), and ketones (21.3%) and esters (11.9%) had the highest expression in QYX (Figure 2a).

To further understand the intrinsic variation in the volatile components detected among the three jujube cultivars, OPLS-DA and HCA were performed on the 66 VOCs. OPLS-DA is a supervised model that reduces systematic noise and extracts variable information with enhanced classification capabilities. It has been successfully used to discriminate between different varieties of fruit, green tea, and sea cucumber [39,40,41]. Using the OPLS-DA model (Figure 2b), it was possible to clearly distinguish between the dried jujube samples according to cultivar with high predictability, a strong goodness of fit (R^2^X = 0.902, R^2^Y = 0.993, and Q^2^ = 0.986), and no overlap. Hierarchical clustering analysis (Figure 2c) indicated that these three jujube cultivars were clustered into two groups according to the accumulation of VOCs. One group included QYX, and the other comprised HZ and HMZ. Thus, together, the OPLS-DA and HCA results suggest that these three jujube cultivars have distinct aroma profiles.

### 3.3. Key Flavor VOCs of Different Jujube Cultivars

The formation of aroma characteristics is not only associated with the concentration of volatile compounds but also with their threshold values. The ROAV is frequently used to evaluate the contribution of VOCs to the overall aroma of samples [26]. In this work, the thresholds of volatiles in water and their aroma types were taken from the relevant literature [13,14,42,43], and we calculated the ROAVs of the VOCs in the dried jujubes (Table 1).

The total of 12 VOCs with ROAV ≥ 0.1, mainly aldehydes, had a great impact on the aroma of dried jujube fruits. Of the twelve VOCs, (E)-2-nonenal, (E)-2-decenal, nonanal, heptanal, decanal, and octanal had an ROAV > 1, indicating that these were the key aroma volatiles of dried jujubes. 1-octen-3-ol, hexanal, (E)-2-hexenal, benzeneacetaldehyde, (E)-2-octenal, and 2-pentyl-furan had ROAVs ranging from 0.1 to 1 and were the modifying components of dried jujube aroma. Working according to previous studies, nonanal, 1-octen-3-ol, hexanal, (E)-2-octenal, and 2-pentylfuran were also regarded as critical aroma-active compounds that provide the typical aromas tof *Jinsixiaozao*, *Youzao*, *Yuzao*, and *Junzao* [14,42]. Different cultivars of dried jujubes have different ROAVs of volatile components. Specifically, the ROAVs of (E)-2-nonenal, octanal, nonanal, and 1-octen-3-ol in HMZ were the highest, standing at 100, 25.11, 16.84, and 2.36, respectively. Nonanal, octanal, and (E)-2-nonenal exhibited “lemon, orange, green, floral and fatty” aromas and were considered the key volatiles of apricots, grapes, and walnut [44,45,46]. 1-octen-3-ol is the predominant aroma volatile of mushrooms [47]. The highest ROAV of (E)-2-decenal (ROAV = 100) was observed in HZ and QYX. (E)-2-decenal, with a “fatty, tallow and chicken fat” aroma, is the main aroma substance of milk, macadamia nuts, and Amomum tsao-ko [48,49,50].

Overall, the differential accumulations of these key flavor VOCs in different jujube cultivars make their aromas distinctive. HMZ displays a higher accumulation of key aroma components and has a better aroma than HZ and QYX.

### 3.4. Metabolite Profiles of Different Jujube Cultivars

**Figure 3:**
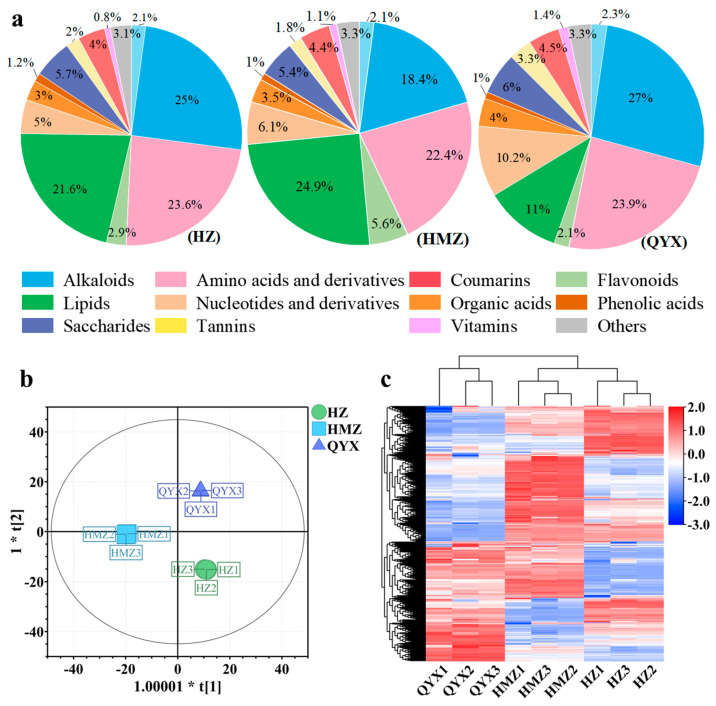
(**a**) Pie chart showing proportions of 15 metabolite categories. (**b**) OPLS-DA score plots. (**c**) HCA of metabolites of different dried jujube cultivars.

The categories and contents of metabolites in jujube fruit directly affect its nutritional and developmental value. Our comprehensive analysis showed that dried jujube is rich in carbohydrates, flavonoids, polyphenols, vitamins, proteins, polysaccharides, and nucleotides, possessing high nutritional and biological value [15]. In this work, a total of 454 metabolites were identified and classified into 15 classes, (Table S2), including 83 lipids, 56 amino acids and derivatives, 53 flavonoids, 50 alkaloids, 44 organic acids, 31 nucleotides and derivatives, 34 phenolic acids, 30 terpenoids, 20 saccharides, 13 vitamins, 7 alcohols, 7 tannins, 6 coumarins, 2 steroids, and 18 other compounds. The total of the items identified was much higher than in a previous study [22]. As shown in Figure 3a, the alkaloids, amino acids and derivatives, flavonoids, lipids, nucleotides and derivatives, saccharides, and terpenoids are the main nutrients in dried jujube fruits, accounting for 84.72–87.77% of the total content, which is consistent with a previous report [51].

To better understand the metabolomic differences among the three jujube cultivars in Xinjang, a multivariate analysis of the 454 metabolites was carried out. As shown in Figure 3b, the dried jujube samples could be clearly distinguished according to cultivar using the OPLS-DA model, without any overlap (R^2^X = 0.89, R^2^Y = 0.999, and Q^2^ = 0.996). Using HCA (Figure 3c), these three jujube cultivars were mainly observed to cluster into two groups according to their accumulation of metabolites. The first group included QYX and the second group comprised HZ and HMZ. The above results of the multivariate statistical analysis suggest that the three jujube cultivars have distinct metabolite profiles.

Related studies have shown that the metabolic characteristics of different cultivars are closely related to their genetic relationship [52,53,54]. In this work, the results of multivariate analysis of VOCs and metabolites indicate that HZ and HMZ are closely related, and that both are distantly related to QYX (Figure 2 and Figure 3), which differs from previous findings at the genomic level [4]. This may be due to the environmental conditions faced after their introduction to Xinjiang altering the metabolism of the jujubes, resulting in a differential accumulation of volatiles and metabolites. Previous research demonstrated that the geographical and climatic conditions in different regions affect the accumulation of metabolites in radix scrophulariae and black wolfberry fruit, causing samples of the same cultivars to exhibit different metabolic properties in different habitats [55,56]. Like metabolites, the levels of VOCs in plants are also highly susceptible to the environment in which plants are cultivated. A study suggested that different production areas contributed to the distinctive aromatic profile of prickly ash pericarps and that the soil factors (Pb, Nt, Pt, As, and Mn) significantly influenced the VOC profiles of the samples [57]. Recent studies, carried out on wheat varieties grown in different locations, have shown that the environmental conditions affected the VOC fingerprint of the wheat much more than the cultivar itself [58,59]. Therefore, the effects of natural environmental conditions on jujube fruit volatiles and non-volatile metabolites need to be further explored in future studies, and their potential environmental influences need to be elucidated.

### 3.5. Differential Metabolites among Different Jujube Cultivars

**Figure 4:**
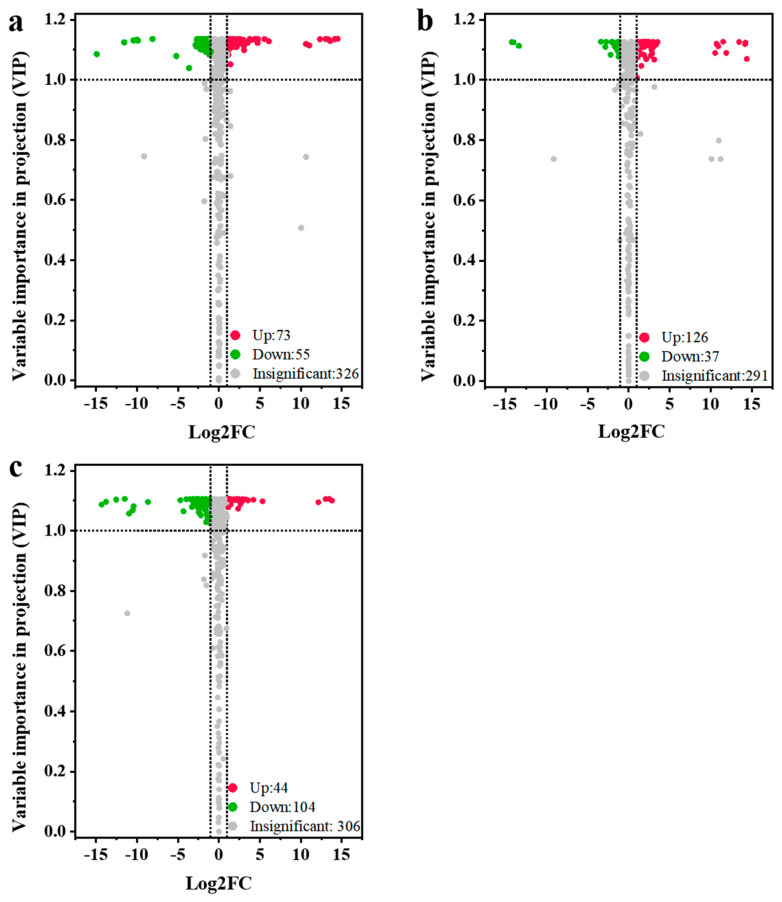
Volcano plots for differential metabolites among different dried jujube cultivars. (**a**) QYX vs. HZ, (**b**) QYX vs. HMZ, and (**c**) HMZ vs. HZ.

To further reveal the accumulation differences in metabolites among different jujube cultivars, the three jujube varieties were compared in pairs, including QYX vs. HZ, QYX vs. HMZ, and HMZ vs. HZ. There were 128 differential metabolites (73 upregulated and 55 downregulated) between QYX and HZ (Figure 4a), 163 differential metabolites (126 upregulated and 37 downregulated) between QYX and HMZ (Figure 4b), and 148 differential metabolites (44 upregulated and 104 downregulated) between HMZ and HZ (Figure 4c). Combining the three comparisons, a total of 237 metabolites showed differential accumulation in at least one pairwise comparison (Table S3), including 62 lipids, 37 flavonoids, 27 alkaloids, 20 nucleotides and derivatives, 17 terpenoids, 15 organic acids, 12 amino acids and derivatives, 12 phenolic acids, 8 saccharides, 7 tannins, 6 coumarins, 3 vitamin, 2 alcohols, and 9 other metabolites. This indicates that lipids, flavonoids, alkaloids, and nucleotides are the key metabolites responsible for the differences in the metabolic profiles among the three jujube cultivars. Detailed comparisons are shown below.

#### 3.5.1. Lipids

A total of 62 lipids were differentially expressed among the 3 jujube cultivars, with HMZ having the highest content and QYX having the lowest. Notably, HMZ had the highest content of 38 lipids, including 9-KODE, DGMG (18:1), FA (22:7), LysoPC (14:0), LysoPE (14:0), PC (35:4), MAG (18:2), isomer 1, and so on. Meanwhile, only five lipids—FA (20:2), FA-OH (18:2), lauric acid C (12:0), linolenic acid ethyl ester, and octadecatrienoic acid methyl ester—exhibited their highest contents in QYX. Lipids not only provide the energy needed for plant life activities, but they also act as precursors to volatile compounds. A previous study showed that fatty acids can produce volatile substances, such as aldehydes, esters, ketones, and alcohols, through the α-oxidation, β-oxidation, and LOX pathways, further affecting fruit flavor [60]. Therefore, it is speculated that the high contents of lipids and differential lipids in HMZ can generate more VOCs, leading HMZ to have a richer aroma, which is consistent with the results of testing the volatile compounds in different cultivars of dried jujube, mentioned above.

#### 3.5.2. Flavonoids and Alkaloids

Flavonoids and alkaloids, which are the main nutritional active ingredients of dried jujube fruit, can provide important nutrients to the human body, inducing pharmacological effects [1,61]. In this work, 26/37 differential flavonoids, such as cyanidin, cymaroside, keracyanin, etc., exhibited the highest contents in HMZ. These flavonoids were 1.33 to 18,333.33 times more expressed than those in QYX and 1.12 to 8.68 times more expressed than those in HZ. Additionally, out of the 27 differential alkaloids, 13 had the highest level of accumulation in QYX, such as zizyphusine neohesperidose, lysicamine, nuciferine, and so on, which were 1.15 to 6.37 times more expressed than those in HZ and 2.08 to 10.37 times more expressed than those in HMZ.

This suggests that HMZ can be used as a supplementary food for related nutrients, and that QYX has greater potential for the development of alkaloid medicines.

#### 3.5.3. Nucleotides and Derivatives

Of these 20 nucleotides, 9, 7, and 4 were the most abundant in QYX, HMZ, and HZ, respectively. Notably, both adenosine 3′,5′-cyclic monophosphate (cAMP) and guanosine 3′,5′-cyclic monophosphate (cGMP) exhibited the highest content in QYX, which was 2.32–2.82 times higher than the level found in HMZ and HZ. cAMP and cGMP are the main cyclic nucleotides in jujube fruits. cAMP is also the most characteristic bioactive substance in jujube fruit, in which its content is 2000 times higher than that of pears, peaches, and other fruits [62]. These attributes grant jujubes various healthcare functions, such as improving liver function and myocardial hypoxia, acting as an anti-fatigue agent, and inhibiting cell carcinogenesis [1].

#### 3.5.4. Other Metabolites

A total of 17 terpenoids with differential expression profiles, 12 differentially expressed amino acids, 12 phenolic acids with differential expression profiles, and 27 other compounds were identified among the 3 jujube cultivars.

Among them, 12/17 differential terpenoids, L-(+)-arginine, and H-homoarg-OH (amino acids and derivatives), half of the phenolic acids, and procyanidin B2, procyanidin B3, and procyanidin A3 exhibited the highest levels in HMZ.

HZ had the highest contents of L-glutamic acid, L-glutamine, and oxidized glutathione (amino acids and derivatives). Meanwhile, the other half of the phenolic acids, such as cinnamic acid, hydrocinnamic acid, etc., were the most abundant in HZ.

Finally, 7 other compounds, namely scopoletin, nicotinate ribonucleoside, and so on, were highly expressed in QYX.

Taken together, the results suggest that HMZ is rich in flavonoids, triterpenes, organic acids, and lipids, giving it wide potential for use in drug development [63,64,65]. QYX is rich in nucleotides and alkaloids. HZ contains more amino acids and saccharides. These differences in the metabolite accumulation of the dried fruits of the different jujube cultivars give them different flavor and nutritional properties, providing a basis for the further development and utilization of dried jujube fruits.

### 3.6. KEGG Pathway Enrichment Analysis of the Differential Metabolites

**Figure 5:**
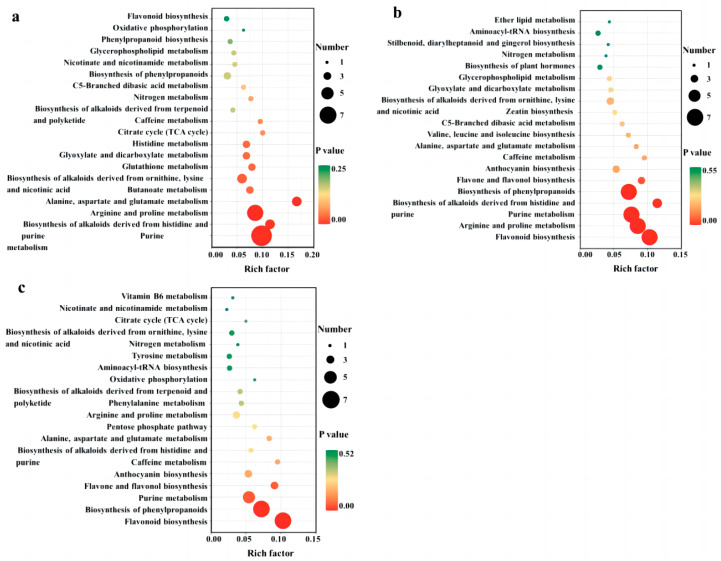
KEGG pathway enrichment analysis of differential metabolites among different dried jujube cultivars. (**a**) QYX vs. HZ, (**b**) QYX vs. HMZ, and (**c**) HMZ vs. HZ.

After identifying the differential metabolites among cultivars using pairwise contrasts, all of the associated metabolic pathways were determined using KEGG analysis. The results of the differential metabolic pathways with the top 20 *p*-values are displayed in Figure 5a–c. We found differential metabolites between QYX and HZ in 40 metabolic pathways (Figure 5a), among which purine metabolism, arginine and proline metabolism, alanine, aspartate, and glutamate metabolism, the biosynthesis of alkaloids derived from histidine and purine, the biosynthesis of alkaloids derived from ornithine, lysine, and nicotinic acid, butanoate metabolism, glutathione metabolism, glyoxylate and dicarboxylate metabolism, and the histidine metabolism pathway showed significant differences (*p* < 0.05). In the comparison of QYX vs. HMZ (Figure 5b), a total of 47 different pathways (5 of them being significant) were observed, including the biosynthesis of phenylpropanoids, purine metabolism, flavonoid biosynthesis, arginine and proline metabolism, purine metabolism, the biosynthesis of phenylpropanoids, and the biosynthesis of alkaloids derived from histidine and purine. In comparing HMZ to the HZ (Figure 5c), it was found that differential metabolites were involved in 42 metabolic pathways. Among these, flavonoid biosynthesis, the biosynthesis of phenylpropanoids, and the purine metabolism pathway were significantly different.

All three sets of enrichment analyses showed that purine metabolism, flavonoid biosynthesis, flavone and flavanol biosynthesis, and the biosynthesis of phenylpropanoids were the significantly different pathways responsible for the metabolic differences among the three jujube cultivars. This is consistent with the results of the differential accumulation of metabolites.

## 4. Conclusions

In this work, we systematically investigated the soluble sugars, organic acids, volatiles, and metabolites present in three jujube cultivars in order to compare the flavor and nutritional variations among them. This work provided comprehensive information regarding the composition and abundance of volatile and non-volatile metabolites in the three major cultivated jujube cultivars in Xinjiang, thus providing references to enable their further exploitation. The results revealed that the main soluble sugar in dried jujube fruit is sucrose; the main organic acid is malic acid; the key volatile aroma compounds are (E)-2-nonenal, (E)-2-decenal, heptanal, decanal, nonanal, and octanal; and the main nutrients are alkaloids, amino acids, flavonoids, lipids, nucleotides, terpenoids, organic acids, and saccharides. These three jujube varieties have distinct flavors and nutritional characteristics, as well as many kinds of metabolites. HZ has the highest sugar content and a sweeter taste, HMZ has the highest accumulation of key aroma substances and the best aroma, and HMZ and QYX have higher accumulations of bioactive metabolites and, thus, possess health-promoting value.

## Figures and Tables

**Table 1:** The ROAVs of VOCs in three dried jujube cultivars.

VOC	Threshold (μg/kg) *^a^*	Odor Type *^b^*	HZ		HMZ		QYX	
			Relative Content %	ROAV	Relative Content %	ROAV	Relative Content %	ROAV
Heptanoic acid	3000	Rancid, sour, cheesy, and sweat	0.006	0.000	0.012	0.000	0.013	0.000
Tetradecanoic acid	10,000	Faint waxy, fatty, pineapple, and citrus peel	47.668	0.047	37.179	0.062	42.414	0.039
Dodecanoic acid	10,000	Mild fatty and coconut bay oil	3.511	0.003	1.097	0.002	2.078	0.002
n-Hexadecanoic acid	2000	Slightly waxy and fatty	7.755	0.038	9.221	0.077	16.532	0.076
Octanoic acid	910	Fatty, waxy, rancid, vegetable, and cheesy	0.219	0.002	0.204	0.004	0.179	0.002
Butanoic acid	1400	Dairy-like, cheesy, buttery, and fruity	0.094	0.001	0.134	0.002	0.079	0.001
n-Decanoic acid	10,000	Unpleasant, rancid, and sour	0.002	0.000	0.076	0.000	0.060	0.000
Pentanoic acid	280	Sour milky, tobacco, and fruity	0.026	0.001	0.168	0.010	0.014	0.000
1-Octen-3-ol	1	Mushroom, vegetative, earthy, and oily	0.092	0.902	0.141	2.361	0.043	0.392
(E)-2-Nonenal	0.08	Fatty, green, citrus, cucumber, and melon	0.458	56.318	0.479	100.00	0.158	18.175
(E)-2-Decenal	0.3	Fatty, earthy, green, cilantro, and fat tallow	3.049	100.00	0.919	51.163	3.252	100.00
Hexanal	4.5	Fresh, green, fatty, and fruity (apple, citrus, and orange)	0.013	0.028	0.052	0.192	0.083	0.170
Nonanal	1	Fruity, floral, and fatty (rose, orange, melon, nutty, and coconut)	0.661	6.499	1.009	16.840	0.126	1.161
Benzaldehyde	320	Strong sharp bitter almond and woody	0.089	0.003	0.108	0.006	0.032	0.001
(Z)-2-Heptenal	13	Apple and vegetable	0.017	0.013	0.051	0.066	0.007	0.005
Heptanal	3	Fresh, fatty, and green herbal	0.879	2.882	1.242	6.913	1.292	3.973
(E)-2-Hexenal	17	Green, banana, cheesy, and vegetative	0.027	0.016	0.098	0.096	0.147	0.080
Decanal	0.1	Sweet, orange peel, citrus floral, and green melon	0.017	1.712	0.026	4.291	0.011	1.042
Benzeneacetaldehyde	4	Honey, sweet, floral, chocolate, and spicy	0.088	0.216	0.146	0.609	0.060	0.139
(E)-2-Octenal	3	Fresh, green, fatty, cucumber, herbal, and banana	0.000	0.001	0.014	0.080	0.005	0.014
Octanal	0.7	Citrus peel, green herbal, fresh, and fatty	0.377	5.305	1.053	25.111	0.230	3.030
Furfural	282	Sweet, nutty, caramellic, almond, and baked bread	0.009	0.000	0.097	0.006	0.020	0.001
Hexadecanoic acid ethyl ester	2000	Mild fruity, creamy, balsamic, and greasy	0.167	0.001	1.427	0.012	0.744	0.003
3-Octanone	28	Fresh, sweet, herbal, lavender, and mushroom	0.158	0.056	0.162	0.097	0.672	0.222
5-ethyldihydro-2(3H)-Furanone	1600	Sweet, creamy, tobacco, and green coconut	0.423	0.003	0.557	0.006	1.353	0.008
6-methyl-5-Hepten-2-one	100	Fruity, apple, musty, creamy, slight cheesy, and banana	0.254	0.025	0.084	0.014	0.173	0.016
2-pentyl-Furan	6	Fruity, green, bean, and vegetable	0.052	0.086	0.109	0.303	0.036	0.056

Note: ***^a^***—The threshold of volatile compounds in water taken from the literature [13,14,42,43]. ***^b^***—The odor type of volatile compounds was obtained from the TGSC Information System (http://www.thegoodscentscompany.com, accessed on 12 June 2023) and several studies [13,14,43].

## Data Availability

The original contributions presented in the study are included in the article/Supplementary Materials, further inquiries can be directed to the corresponding author.

## Supplementary Materials

The following supporting information can be downloaded at: https://www.mdpi.com/article/10.3390/foods13081193/s1. Table S1: The list of 66 volatile components detected in three dried jujube fruits; Table S2: The list of 454 metabolites detected in three dried jujube fruits; Table S3: The list of 237 differential metabolites identified among three dried jujube fruits.
